# What is the impact of nicotine pouches on oral health: a systematic review

**DOI:** 10.1186/s12903-024-04598-8

**Published:** 2024-08-03

**Authors:** Dulyapong Rungraungrayabkul, Piyada Gaewkhiew, Tippanart Vichayanrat, Binit Shrestha, Waranun Buajeeb

**Affiliations:** 1 Department of Oral Medicine and Periodontology, Faculty of Dentistry, Mahidol University, 6 Yothi Street, Ratchathewi, Bangkok, 10400 Thailand; 2 Department of Community Dentistry, Faculty of Dentistry, Mahidol University, Bangkok, 10400 Thailand; 3 Department of Prosthodontics, Faculty of Dentistry, Mahidol University, Bangkok, 10400 Thailand; 4 WHO Collaborating Centre for Oral Health Education and Research, Faculty of Dentistry, Mahidol University, Bangkok, 10400 Thailand

**Keywords:** Nicotine pouch, Oral health, Systematic review

## Abstract

**Background:**

Increase in nicotine pouch (NP) users, particularly among the young, is a matter of concern requiring a comprehensive understanding of its short- and long-term oral health implications. The objective of this research was to systematically review potential oral side-effects associated with NP usage.

**Methods:**

This systematic review was conducted following the PRISMA guidelines. Databases (Medline via PubMed, Scopus, Cochrane Trial, and Google Scholar) were searched for relevant studies up to February 2024. Modified Newcastle-Ottawa Scale (NOS) and the Risk Of Bias In Non-randomized Studies - of Exposure (ROBINS-E) tool were used to assess the quality and bias of the included studies.

**Results:**

Three studies were included for this review, two from Europe and one from USA, and considered of a total of 190 participants. All studies were deemed to have a high risk of bias. Participants used NP for periods ranging from 1 month to 10 years. Among these studies, only one study provided information on the usage pattern between 1 and 5 units for an average of 11 ± 7 min per session. Oral mucosal changes at the site of placement were common among NP users. Oral lesions varied from slight wrinkling to various white lesions, seemingly related to the NP units consumed per day and their duration of usage. Other oral side effects included dry mouth, soreness, gingival blisters, and a strange jaw sensation.

**Conclusions:**

Research on the use of NP and its effect on oral health are currently limited. The use of NP should take into consideration the short-and-long-term effects, especially on oral health. Further studies are crucial to understand oral health implications associated with NP usage.

**Systematic review registration:**

PROSPERO Registration number CRD 42,024,500,711.

**Supplementary Information:**

The online version contains supplementary material available at 10.1186/s12903-024-04598-8.

## Background

There has been a global decline in smoking tobacco consumption in recent years [1]; nevertheless, its use still results in more than 7 million annual deaths [2]. Furthermore, manufacturers have introduced a wide range of newer non-combustible nicotine products into the market that includes e-cigarette, nicotine pouch (NP), and dissolvable tobacco product. They are also available in an array of flavors making them appealing to new young experimental users. Since their introduction in the United States in 2016, NP has been gaining popularity. The sales of NP have risen from 0.6 million units in 2016 to 46 million units in the first half of 2020 [3]. Furthermore, according to a study conducted in 2021, 1/3rd of American adults were reported to be aware of the product [4].

NP is generally placed between the lips and gums similar to Swedish snus for 30–60 min, which allows nicotine to be absorbed systemically via the oral mucosa. Unlike snus, it is devoid of tobacco leaf and constitutes a nicotine-containing cellulose matrix inside a fiber pouch, and added nicotine can be either naturally or synthetically sourced. Moreover, in many countries there is an absence of proper regulation regarding NP, and most consumers may be unaware of the composition, concentration, and purity of nicotine. Particularly of safety concern is the presence of tobacco-specific nitrosamines (TSNAs)- Nitrosonornicotine (NNN), N-nitrosoanatabine (NAT), N-nitrosoanabasine (NAB), and 4-(methyl nitrosamino)-1-(3- pyridyl)-1-butanone (NNK), which may be present in nicotine derived from tobacco leaf. These alkaloid carcinogens, especially NNN, are considered as a risk factor for oropharyngeal carcinoma [5]. In an analysis of 44 NPs by Mallock et al., [6] nicotine contents of the products ranged from 1.79 to 47.5 mg/pouch. TSNAs were also detected in more than half of the 44 NP products with the highest concentrations of NNN and NNK measuring 12.9 ng and 5.4 ng/pouch, respectively [6]. It is also of concern that only a few studies have investigated the safety and efficacy of these products. Oral lesions have been frequently reported with snus-users [7], with histological studies revealing higher levels of proinflammatory cytokines [8]. The presence of these lesions has been associated with higher alkalinity of these products and due to the release of nicotine. Additionally, close approximation of NP to oral tissues has been observed to exacerbate oral lesions [9]. Studies have also shown that nicotine can directly or indirectly be detrimental to periodontal tissue. In vitro studies have demonstrated that nicotine exposure significantly activated nicotinic acetylcholine receptor expression, repressed periodontal ligament fibroblasts cells, and stem cell viability, and increased the generation of cellular reactive oxygen species, subsequently leading to DNA damage and cell death [10–12]. Nicotine has also been associated with increased bone loss through miRNA mechanism [13]. The inclusion of flavoring agents, such as methanol, in NP adds to the safety concern as methanol can potentially amplify the absorption of NNN and nicotine through the buccal mucosa, increasing the health risk. Furthermore, the presence of trace amounts of formaldehyde and chromium has also been identified in some instances [14].

Due to the distinct form, convenience, and composition of NPs in comparison to other smokeless forms of tobacco, such as chewing tobacco and snus, they are marketed as potentially safer alternatives. However, the current level of evidence is insufficient to recommend their use. Considering the increasing popularity of NP among young users, it is crucial to thoroughly examine and understand the short- and long-term effects of NP on oral health. This comprehensive systematic review aimed to provide insights into the potential oral health implications of NP use.

## Methods

This systematic review followed the Cochrane recommendation. The review protocol was registered in PROSPERO (Registration number CRD 42,024,500,711).

### Criteria for considering studies for this review

The broad criteria were predefined to select articles for inclusion, following PICO (Participants, Intervention, Comparison, and Outcome) format. Only cross-sectional, case-control, cohort, and trial studies in humans were included. Case report/series, expert opinion, animal studies, and laboratory works were excluded. Participants or population were any age group without restriction of setting. Interested intervention or exposure was NP. The outcome measurements were oral health status including tooth/teeth, gingiva, oral mucosa, and related sign or symptom within the oral cavity.

### Study selection and data extraction

The search process for the selection of research studies followed the Preferred Reporting Items for Systematic Reviews and Meta-Analyses (PRISMA) guidelines. Four electronic databases were systematically searched: PubMed, Scopus, the Cochrane Library, and Google Scholar up to February 2024 using a combination of Medical Subject Headings (MeSH) terms and text words in two main topics: the exposure (NP usage) and the outcomes (oral health status, tooth status, oral mucosal lesion). Search terms were chosen based on the team expertise and previous related reviews. Only English and Thai language were included. Search strategies are shown in Supplementary 1.

All references retrieved were managed on Rayyan. Duplicated articles were excluded at this stage. An eligibility criterion was used to select the papers from title and abstract by three independent reviewers (TV, PG, DR). In case of disagreements, the reviewers discussed with a fourth author (WB) to obtain a consensus. The full text of publications were sought if at least one of reviewers considered the study was potentially included in the review. An additional manual search was conducted on the references lists of the retrieved studies.

Excel (Microsoft Corp) files were created listing all the retrieved studies with the title, authors, journal, publication year, and reason for exclusion. For eligible studies, four reviewers additionally extracted information on the details, including study design, participants’ characteristics, exposure variable, outcome measurements, covariates/confounders, data analysis, main findings, and notes. Disagreements were resolved through discussion.

### Inter-rater reliability

Inter-rater reliability for screening of titles and abstracts was piloted among the three reviewers (TV, PG, DR) with the first 3 papers and was rated at 100%. Any conflicts were resolved via discussion to arrive at a consensus, or via consultation with a fourth reviewer. Each section of data extraction demonstrated a very high inter-agreement reliability, between reviewer one and two.

### Quality and risk of bias assessment in individual studies

Three reviewers (TV, PG, DR) independently assessed the quality of the included full-text study (*n* = 3). Quality assessment was evaluated using the modified Newcastle Ottawa Scales (NOS) checklist composed of three domains: selection, comparability, and outcomes. For selection, a score was given when the sample was truly or somewhat representative of the community. A good quality scored required 3–4 stars in selection domain AND 1–2 scores in comparability domain AND 2–3 scores in outcome domain; a fair quality study required 2 stars in selection domain AND 1–2 stars in comparability domain AND 2–3 scores in outcome domain; and a poor-quality study 0–1 score in selection domain OR 0 score in comparability domain OR 0–1 score in outcome domain. For risk of bias assessment, the Risk Of Bias In Non-randomized Studies - of Exposure (ROBINS-E) tool was used to assess all included papers across seven different domains including Domain 1: Risk of bias due to confounding; Domain 2: Risk of bias arising from measurement of the exposure; Domain 3: Risk of bias in selection of participants into the study (or into the analysis); Domain 4: Risk of bias due to post-exposure interventions; Domain 5: Risk of bias due to missing data; Domain 6: Risk of bias arising from measurement of the outcome and Domain 7: Risk of bias in selection of the reported result.

### Data synthesis

A meta-analysis of the findings including forest plot and funnel plot could not be performed as the heterogeneity across the included studies was high. Thus, the authors worked on narrative analysis of the findings. The summary table was created to summarize the methodology in each paper, along with quality assessment and risk of bias evaluation based on NOS and the ROBINS-E tool.

## Results

**Fig. 1:**
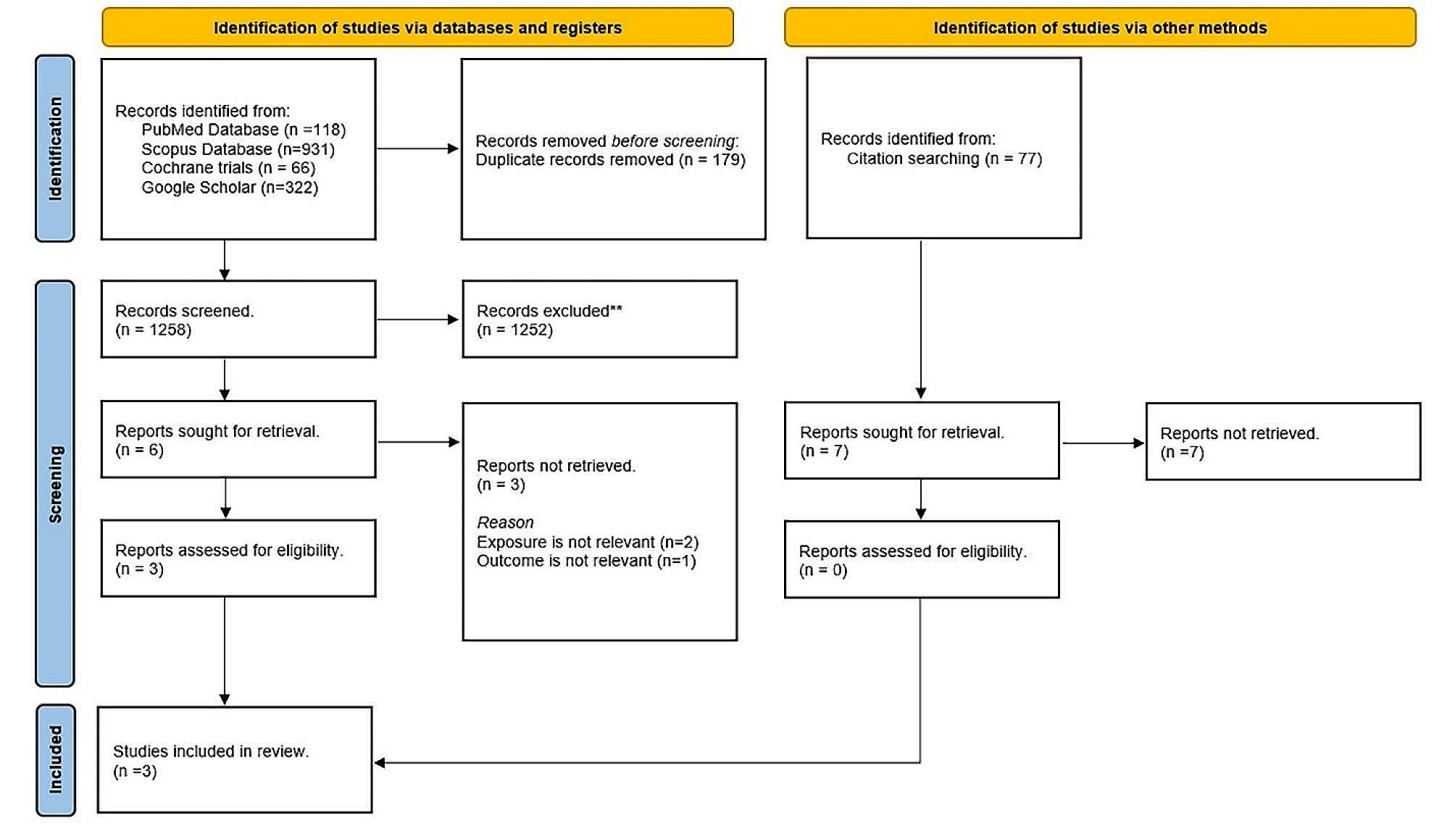
PRISMA flowchart for systematic review

A flowchart of screening and selection studies was shown in Fig. 1. Out of the retrieved 1,258 unique articles, 1,252 articles were excluded after screening titles and abstracts as clearly irrelevant. For the full paper review, three out of six papers were excluded due to irrelevant exposure or outcomes. Finally, three articles, published between 2022 [15, 16] and 2024 [9], were included for analysis. Among them, two were cross-sectional studies [9, 15], while one was a prospective experimental study [16]. Characteristics of the included studies and outcome of oral NP usage were demonstrated in Table 1.

**Table 1:** Characteristics of the included studies and outcome of nicotine pouch usage

Authors **(year)**	Type of Study	Country	Number of participants	Sex	Age (years) Mean ± SD Range	Frequency Mean ± SD Range & duration of usage	Outcome				Note
							Oral mucosal changes	Dental & gingival status	Adverse effects	Salivary biomarkers	
Dowd et al. (2024)	Cross-sectional	USA	118	M = 40 F = 78	30 ± 10 18–67	2 ± 1 NP/session (1–5 NP, Mostly with tobacco-cigarette and/or e-cigarette) Average session time 11 ± 7 mins Mean 13 ± 6 days	Oral lesions	-	- Sore mouth - Sore throat - Upset stomach - Nausea - Strange jaw sensation	-	Retrospective self-reported data Past 30-day NP use
Alizadehgharib et al. (2022)	Prospective	Sweden	60 (57 completed)	M = 39 F = 21	31 ± 10	Ratio NP/total nicotine product = 0.84 For 6 weeks	Clinical four-grade severity scale (1–4) Changes from previous lesions (0–4) - Increased score = 6 - No score change = 12 - Reduced score = 39	- DMFT = 3.7 ± 4.6 (at the beginning) - Gingival retraction 54–57% (no change) - Gingival blisters	- Nausea - Dry mouth - Dizziness	-	Substituted regular Swedish snus users (≥ 2 snus cans for > 1 year) with NP
Miluna et al. (2022)	Cross-sectional	Latvia	12	M = 10 F = 2	25.08	Snus and/or NP user = 12 For 2–10 years	9/12 with oral lesions at upper anterior and premolar areas - White, localized and leathery - White grainy lesion - White linear or round lesions	-	-	-Increased levels of IL-1, IL-6, IL-8, TNF-alpha and LRG1 -IL-6 correlated with mucosal change	Swedish snus and NP group

SD standard deviation, NP Nicotine pouch, M Male, F Female, DMFT Decayed, Missing, filled Teeth, IL Interleukin, TNF tumor necrosis factor, LRG1 leucine-rich alpha-2-glycoprotein 1

### Participants

All the included studies did not report sample size calculation. The number of participants varied between 12 [15] and 118 [9], with a total of 190 participants (Table 1). Age criteria were considered in two studies. Miluna et al. [15]. required participants to be aged 18–35 years, while Dowd et al. [9] required participants to be ≥ 18 years. The participants’ mean age in the included studies were shown in Table 1.

Systemic health and medication usage were included as exclusion criteria in two studies. Miluna et al. [15] excluded participants with systemic diseases or medical conditions and those who were taking daily medication. Similarly, Alizadehgharib et al. [16] excluded participants with diagnosed hypertension, cardiovascular disease, or other medical conditions affecting nicotine metabolism. They also excluded those who had undergone surgery within the last 6 months or had allergies to composite materials. Both studies excluded pregnant participants and those who had used antibiotics, with Alizadehgharib et al. [16] considering antibiotic use within 4 weeks prior to the study initiation and Miluna et al. [15] considering use within at least 6 months. Moreover, Alizadehgharib et al. [16] implemented participant restrictions during the study, which included refraining from tooth cleaning for 72 h, tooth brushing for 48 h, and abstaining from eating and drinking within the last 2 h prior to the visit.

### Use motive/ dependence/ usage patterns

Only the study by Dowd et al. [9] reported participant motives and dependence. The most common motivation for NP use was ‘it comes in flavors I like’ (31%). Similarly, participants reported ‘flavor’ (53%) as the most important aspect of NP use, followed by nicotine level (25%) and brand (22%). In this study, a modified version of the Fagerström Test of Nicotine Dependence-Smokeless Tobacco (FTND-ST) was utilized. The authors noted a mean total FTND-ST score of 7, indicating significant dependence.

In terms of usage patterns, participants in the study by Dowd et al. [9] reported using NP for an average duration of 11 ± 7 min, with a significant majority (75%) positioning NP between their lower lip and gingiva. On an average, participants employed 2 ± 1 units of NP per session, although the usage varied between 1 and 5 units. Conversely, Miluna et al. [15] found that a majority of Swedish snus and/or NP users (77%) preferred placing NP between the upper lip and gingiva, typically consuming 5–10 units per day.

### Parameters evaluated

#### Oral mucosal changes

In the study by Alizadehgharib et al. [16], clinical examination of the oral mucosa at the site of pouch placement followed the protocol outlined by Axéll et al. [8], utilizing a 4-degree scale for scoring. Initially, 90% of participants exhibited pre-existing white mucosal lesions at the Swedish snus placement site. Following the replacement of Swedish snus with NP, the prevalence of white mucosal lesions decreased to 70% among the participants. Specifically, the number of participants with lesions of degrees 3 and 4 decreased, while the number of participants with lesions of degrees 0 and 1 increased by the end of the experiment.

Miluna et al. [15] reported that nine out of twelve participants in the group using Swedish snus and /or NP had white lesions (grainy, round, leathery, and linear) in the oral mucosa. They also found that oral mucosal changes were correlated with the duration of tobacco product use per year and the number of tobacco product units used per day. Participants who had used tobacco products for 5–10 years or used 5–10 tobacco units per day showed a tendency towards experiencing oral mucosal changes.

#### Dental and gingival status

Alizadehgharib et al. [16], recorded Decayed, Missing, Filled Teeth (DMFT) only at the beginning of the study. However, gingival retractions were assessed at visits 1, 2, and 3 throughout the study period. They found no changes in the incidence of gingival retraction during the study.

#### Oral adverse effects

No serious adverse events were detected by Alizadehgharib et al. [16]. Only mild intensity events, including dry mouth, and moderate intensity events, such as gingival blisters, were reported.

In the study by Dowd et al. [9], the most frequently reported oral adverse effects were mouth lesions (48%), sore mouth (37%), and strange jaw sensation (1%).

#### Salivary inflammatory biomarkers and leucine-rich alpha-2-glycoprotein 1 (LRG1)

Miluna et al. [15] evaluated salivary inflammation biomarkers, including interleukin (IL)-1, IL-6, IL-8, tumor necrosis factor-alpha (TNF-alpha), and LRG1 detection. The snus and NP group demonstrated the highest levels of salivary inflammation and LRG1 compared to other groups, including cigarettes, e-cigarettes, and control groups. However, they found that only salivary IL-6 levels correlated with tobacco product types and oral mucosal changes.

#### Quality and risk of bias assessment

**Fig. 2:**
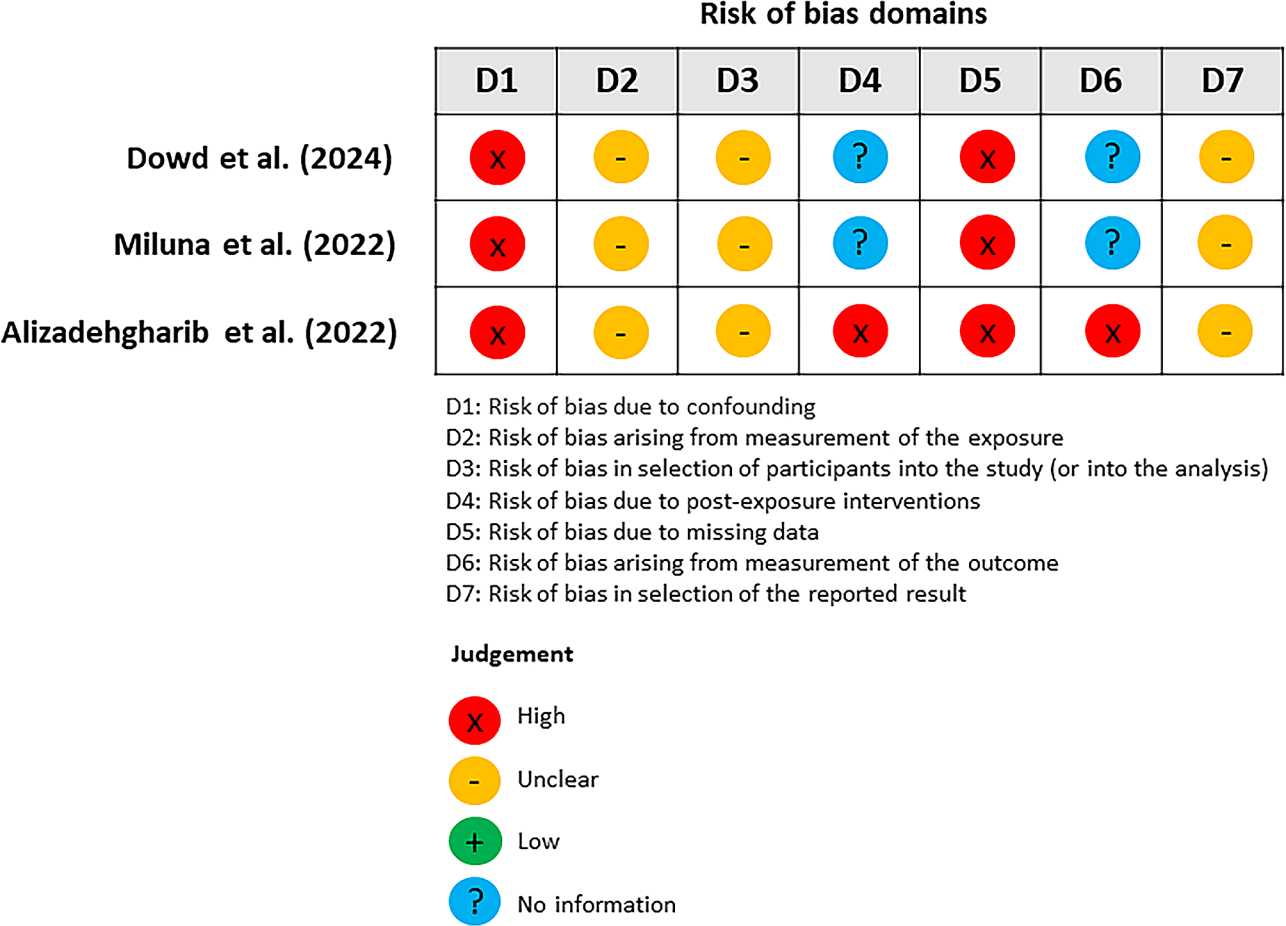
Summary of risk of bias assessed using the ROBINS-E tool

Across three included studies, the overall quality of the evidence and risk of bias assessing NP usage and oral health were presented in Table 2; Fig. 2. For the cross-sectional studies, only the study by Dowd et al. [9] represented the general population and had a response rate over 95%. Moreover, there was no study with sample size more than 400 participants. The prospective study by Alizadehgharib et al. [16], could be scored only for the selection of non-exposed cohort; however, there was no representation of the sample, ascertainment of exposure, and presentation of outcome at the baseline of study. Although all 3 studies failed to report adjustment of variables in their statistical analysis, both cross-sectional studies presented the assessment of the outcome. Only Miluna et al. [15] showed an appropriate statistical analysis with clear description. For the prospective study regarding the outcome module, the study showed adequate follow-up examination and duration.

**Table 2:** Methodological assessment of included studies using Newcastle Ottawa Scales with converting scores

Study- Author (year)		Dowd et al. (2024)	Miluna et al. (2022)		Alizadehgharib et al. (2022)
**NOS items**		Cross-sectional study	Cross-sectional study		Prospective study
**Selection (maximum 3 points)**	**Representativeness of the sample**:	1	0	**Representativeness of the sample**:	0
	**Sample size > 400**	0	0	**Selection of the non-exposed or less-exposed cohort**:	1
	**Non-included subjects/ non-response rate (> 95%)**	1	0	**Ascertainment of exposure**:	0
				**Demonstration that outcome of interest was not present**	0
**Comparability (2 points)**	**Adjustment: age**,** gender**	0	0	**Adjustment: age**,** gender**	0
	**Adjustment: other factors**	0	0	**Adjustment: other factors**	0
**Outcome (3 points)**	**Assessment of the outcome**:	1	2	**Assessment of the outcome**:	0
	**Statistical test**:	0	1	**Time of follow-up**:	1
				**Adequacy of follow-up**:	1
**Total score**		3	3		3
**Overall quality assessment**		High risk of bias	High risk of bias		High risk of bias

Nos Newcastle Ottawa Scales

In addition, the overall of risk of bias assessment following ROBINS-E revealed all studies had high risk of bias which was similar to NOS. Bias in confounding and missing data were high in all studies, whereas measurement of exposure, selection of participants, and selection of reported results were unclear. Post-exposure interventions, arising from the measurement of the outcome, were not assessed in the cross-sectional study, but the risk was higher in the study by Alizadehgharib et al. [16].

## Discussion

This systematic review aimed to describe the effects of NP on oral health, including clinical oral manifestation from the users. Since the NP products are relatively new, the findings from this systematic review indicate limited evidence to confirm the effects of NP on oral health due to the low number of studies and high risk of bias.

Among the three studies included in the systematic review, one was a quasi-experimental study that demonstrated a reduction in oral lesions and inflammatory biomarkers among snus users when snus was replaced with NP, albeit without a control group [16]. It is important to interpret these results cautiously, as the reduction in pre-existing oral lesions does not necessarily imply that NP has no effect on oral lesions. Another cross-sectional study from Latvia could not draw conclusions regarding mucosal changes as snus users and NP (non-tobacco product) users were combined in the same group [15]. Within this cohort, observations revealed various forms of white oral mucosal changes consistent with smokeless tobacco keratosis, a condition that may have the potential to progress to oral squamous cell carcinoma [17]. A third cross-sectional study did not primarily collect oral health outcomes, but it reported self-reported oral lesions, sore mouth, and sore throat as adverse effects among current NP users recruited from online convenience samples [9]. Therefore, none of the included studies could definitively determine the impact of NP on oral health conditions.

Nicotine-containing pouches raise concerns and potential risks for affecting the oral mucosa due to two primary reasons: first, their placement between the lips and gum allows continuous contact with the mucosa, enabling users to use them longer and more frequently than other tobacco or nicotine products, and second, their high nicotine content, which is primarily absorbed via the oral mucosa. One of the studies included in the analysis showed a significant percentage of mucosal changes in the group of users who used NP and snus, particularly among those with longer years of use and frequent consumption of 5 to 10 pouches per day [15]. The presence of mucosal white lesions associated with elevated levels of inflammatory biomarkers such as IL-6, IL-8, IL-1 beta, and TNF-alpha suggests a heightened risk of oral cancer [15, 18, 19]. Specifically, individuals in the NP and snus group exhibited the highest levels of these inflammatory markers. In addition, the highest level of LRG1 was found in the NP and snus users [15]. These findings further emphasized the potential risk for oral cancer development in these populations [20]. Although the study could not distinguish between users of snus and tobacco-free NP, its results suggested that individuals using NP might have a higher tendency to develop oral mucosal white lesions in the areas where these products are placed compared to other forms of tobacco or nicotine delivery systems. While the toxicant levels in NP appear lower than those in snus and other tobacco products [21, 22], numerous NPs contain nicotine levels higher than what is stated on the labels, with specific products reaching nearly 50 mg per pouch [23]. These elevated nicotine levels could potentially result in toxicity and adverse effects on the cardiovascular system, as well as other health-related complications [24]. Due to their convenience, flavor options, and discreet nature, tobacco-free NPs are anticipated to gain popularity [25], especially in countries where they remain largely unregulated [26]. Additional research is crucial, especially with larger sample sizes, and it is imperative to distinguish between snus and NP in these studies.

The detrimental effects of smoking and smokeless tobacco on oral health, particularly periodontal health, are widely recognized [27]; [28]; [29]. However, due to the properties of tobacco products used in earlier studies, research into the direct impact of nicotine on oral and periodontal health has been limited [30]. Gingival blisters were reported in one of our included study [16]. Nonetheless, several animal and in vitro studies have shown that nicotine promotes conditions such as gingivitis [31], periodontal disease [32], and bone destruction [33], supporting the hypothesis that NP may induce inflammation in periodontal tissue. Previous studies suggest that concentrations exceeding 1 mM are more likely to cause inflammation in the oral cavity [34], and the increase of nicotine level significantly associated with higher *S.mutans* and caries severity in rats [35]. These findings highlight the potential influence of nicotine on periodontal tissue and the oral adverse effects on NP users.

Marketing campaigns promoting non-tobacco NP or “novel” nicotine may attract tobacco users [36] and also appeal to experimental users [3, 37], potentially leading to nicotine addiction. Flavorings, similar to other smokeless tobacco products like moist snuff and snus, likely play a significant role in enticing young people to initiate and use NP freely [38, 39]. While NP may offer greater satisfaction compared to other forms of nicotine replacement therapy like lozenges and gum [21], recent studies suggest that NP’s craving relief may not assist in quitting tobacco products and could lead to misuse due to their higher plasma nicotine delivery compared to traditional cigarettes [40]. The high and wide range of unprotonated nicotine content in NP could facilitate users’ transition to products with progressively higher nicotine levels, potentially exacerbating addiction, and resulting in continuous exposure to other harmful tobacco substances. Additionally, recent studies indicated that some flavors and additive substances in NP may induce toxicological responses during chronic usage [41, 42]. Therefore, policies and regulations on NP contents and flavours is crucial, especially for young individuals and those who use tobacco products or nicotine dependence.

Several limitations of this study include data availability, study quality, and participant and outcome heterogeneity. Additionally, individual usage patterns, such as nicotine concentration, percentage of unprotonated nicotine, number of pouches used, duration, and frequency, also influence nicotine release in NP. Therefore, assessing these variables is necessary to compare oral health outcomes and accurately measure the effects of NP.

This systematic review highlights a knowledge gap and emphasizes the necessity for well-conducted studies to elucidate the attributes of NP, its application methods, and potential misuse contributing to heightened risks of oral health issues in various aspects. Currently, the impact of NP on oral health remains incompletely understood despite their increasing popularity among users of new nicotine products. Future studies are required to deepen our understanding of this topic, offering more informed guidance to health practitioners, patients, general population, and policy makers.

## Conclusion

Although studies on the impact of NP on oral health status are scarce, available evidence showed that NP could affect oral mucosa and gingival status. Malignant potential of the oral lesions is still of concern. Therefore, further studies on short-and-long-term effects on oral health are required.

### Electronic supplementary material

Below is the link to the electronic supplementary material.

Supplementary Material 1

## Data Availability

All data analyzed during this study are included in this published article and its supplementary information file.

## Abbreviations

NP: Nicotine pouch
TSNAs: Tobacco-specific nitrosamines
NNN: Nitrosonornicotine
NAT: N-nitrosoanatabine
NAB: N-nitrosoanabasine
NNK: 4-(methyl nitrosamino)-1-(3- pyridyl)-1-butanone
PICO: Participants, Intervention, Comparison, and Outcome
PRISMA: Preferred Reporting Items for Systematic Reviews and Meta-Analyses
MeSH: Medical Subject Headings
NOS: Newcastle-Ottawa Scale
ROBINS-E: Risk Of Bias In Non-randomized Studies - of Exposure
FTND-ST: Fagerström Test of Nicotine Dependence-Smokeless Tobacco
DMFT: Decayed, Missing, Filled Teeth
LRG1: Leucine-rich alpha-2-glycoprotein 1
IL: Interleukin
TNF-alpha: Tumor necrosis factor-alpha
