# Supplementary material for: What is the impact of nicotine pouches on oral health: a systematic review
Source: BMC Oral Health. 2024 Aug 3;24:889. doi: 10.1186/s12903-024-04598-8 (PMC11297755; doi:10.1186/s12903-024-04598-8)
Supplement: Supplementary file 1 — Supplementary Material 1 [file 12903_2024_4598_MOESM1_ESM.docx]

**RESEARCH QUESTION:**

*Does ‘nicotine pouch’ usage impact on oral health?*

**DATABASE:** PubMed, Cochrane, Scopus, and Google Scholar

**I/E Exposure: Nicotine pouch**

"Tobacco derived nicotine"[Title/Abstract] OR "Oral nicotine product"[All Fields] OR "Tobacco free"[All Fields] OR (("tobacco products"[MeSH Terms] OR ("tobacco"[All Fields] AND "products"[All Fields]) OR "tobacco products"[All Fields] OR "tobacco"[All Fields] OR "nicotiana"[MeSH Terms] OR "nicotiana"[All Fields] OR "tobacco s"[All Fields] OR "tobaccos"[All Fields]) AND "leaf tree"[All Fields]) OR "All white"[Title/Abstract] OR "nicotine pouch"[All Fields]

**O outcome: oral health**

"Oral"[All Fields] OR "Oral cancer"[All Fields] OR "Potentially Malignant lesion"[All Fields] OR "Oral lesion"[All Fields] OR "Oral mucosa"[All Fields] OR "Precancerous"[All Fields] OR "oral ulcer"[All Fields] OR "Oral dryness"[All Fields] OR "Oral soreness"[All Fields] OR "Oral irritation"[All Fields] OR "mouth irritation"[All Fields]

**********************************************************************************************

**
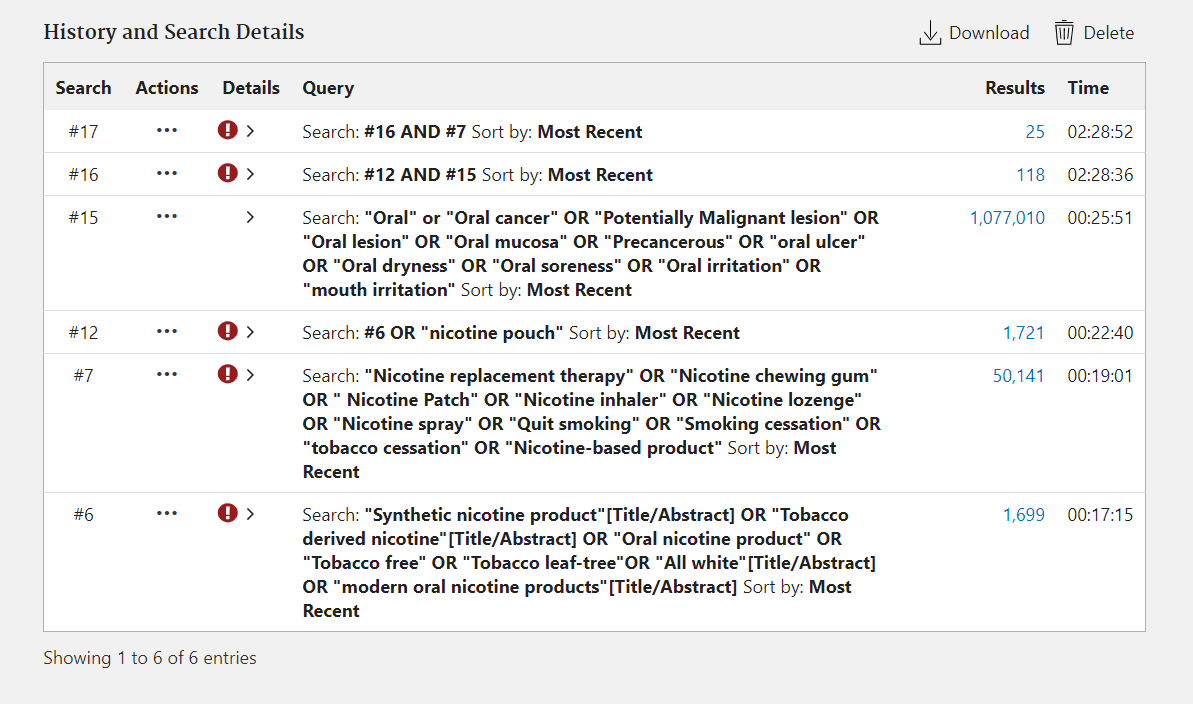
**
